# Changing food availability and its effect on the heritability of offspring size in woodland passerine birds

**DOI:** 10.1111/1365-2656.70204

**Published:** 2025-12-04

**Authors:** Emma Vatka, Markku Orell, Seppo Rytkönen, Juha Merilä

**Affiliations:** ^1^ Research Programme in Organismal and Evolutionary Biology, Faculty of Biological and Environmental Sciences University of Helsinki Helsinki Finland; ^2^ Ecology and Genetics Research Unit, Faculty of Science University of Oulu Oulu Finland; ^3^ Area of Ecology & Biodiversity, School of Biological Sciences The University of Hong Kong Hong Kong Hong Kong SAR

**Keywords:** body size, food availability, growth rate, heritability, long‐term study, *Parus major*, *Poecile montanus*, random regression animal model

## Abstract

1. Climate warming has been associated with widespread body size declines in many vertebrate taxa, but relatively little is known about possible climate warming induced shifts in trait heritabilities.
2. The main goal of the study was to investigate how changing food availability affects evolutionary potential of four traits related to nestlings' body size.
3. We used long‐term, pedigree structured data of two woodland passerines living in the boreal zone, the Willow Tit (*Poecile montanus*) and the Great Tit (*Parus major*), to study how food availability for their nestlings has changed in time, how this has influenced their morphological traits (viz. wing, tail & tarsus length & body mass) and their heritabilities and evolvabilities. This was done by assessing heritabilities under varying food availabilities using random regression animal models.
4. We found that caterpillar food availability had increased over the 25‐year‐long study period and that this was accompanied by increases of nestlings' body mass, but not other morphological traits. All traits were heritable in both species, but additive genetic variance, heritability and evolvability were affected by food availability only in the case of the wing length, being higher under low food availability (the Great Tit) or higher under low and high food availability (the Willow Tit).
5. We conclude that changes in food availability seem to have limited influence on evolutionary potential of body size traits in these two passerine birds.

## Graphical abstract

Vatka et al. investigated how changing food abundance affects evolutionary potential of offsprings' body size traits in two woodland passerines. Food availability increased over the 25‐year‐long study period, accompanied by increases in body mass. Body size traits were heritable, yet only wing length's evolutionary potential was affected by food availability. Photo credit: A. Laine.

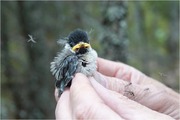

## Tiivistelmä

1. Ilmaston lämpeneminen on yhdistetty monien selkärankaisten eläinten ruumiinkoon pienenemiseen, mutta mahdollisista ilmaston lämpenemisen vaikutuksista ominaisuuksien heritabiliteetteihin tiedetään melko vähän.
2. Tämän työn tavoitteena oli tutkia, kuinka muuttuvat ravinnonsaatavuudet vaikuttavat neljän pesäpoikasten ruumiinkokoa kuvastavan ominaisuuden evolutiiviseen potentiaaliin.
3. Käytimme kahden boreaalisella vyöhykkeellä elävän varpuslinnun, hömötiainen (*Poecile montanus*) ja talitiainen (*Parus major*), pitkäaikaista, sukupuurakenteista aineistoa tutkiaksemme kuinka pesäpoikasaikainen ravinnonsaatavuus on muuttunut ajassa, miten tämä on vaikuttanut pesäpoikasten morfologisiin ominaisuuksiin (siiven, pyrstön ja nilkan pituus sekä ruumiinpaino) ja näiden ominaisuuksien heritabiliteetteihin ja evolvabiliteetteihin. Tämä tehtiin tarkastelemalla heritabiliteetteja vaihtelevissa ravinto‐oloissa käyttämällä nk. random regression animal model ‐mallinnusta.
4. Havaitsimme, että toukkaravinnon saatavuus on parantunut 25 vuoden tutkimusjakson aikana, ja sen myötä pesäpoikasten ruumiinpainot ovat nousseet—muissa morfologisissa mitoissa ei havaittu ajallisia muutoksia. Kaikki tarkastellut ominaisuudet olivat osittain periytyviä kummallakin lajilla, mutta vain siiven pituuden additiivinen geneettinen varianssi, heritabiliteetti ja evolvabiliteetti olivat yhteydessä ravinnon saatavuuteen. Nämä olivat korkeimmillaan, kun ravintoa oli vähän (talitiainen) tai kun ravintoa oli vähän tai erityisen paljon (hömötiainen).
5. Johtopäätöksemme on, että muutoksilla ravinnon saatavuudessa näyttää olevan vain rajallisesti vaikutusta tutkimiemme kahden varpuslintulajin ruumiinkokoa kuvaavien ominaisuuksien evolutiiviseen potentiaaliin.

## 1 INTRODUCTION

To persist and prosper, natural populations need to adapt to anthropogenically induced changes in their environments, such as the warming of the climate. Climate warming has indeed led to a plentitude of responses in the wild, including advancement of phenology in seasonal environments (Parmesan, 2006) and distributional range shifts (Parmesan, 2006; Taheri et al., 2021; Thomas, 2010). Also, changes towards smaller body sizes in a warming world have been frequently observed (e.g. Gardner et al., 2011; Merilä & Lv, 2025; Sheridan & Bickford, 2011; Teplitsky et al., 2008), although these trends are not universal (Merilä & Lv, 2025; Radchuk et al., 2019). These changes can arise either from phenotypic plasticity, evolution or both (Gienapp et al., 2008). However, as individuals can be plastic only to a certain extent, only evolutionary adaptation or migration to more favourable environments is sustainable in the long‐term when the limits of plasticity have been reached (Gienapp et al., 2008; Gienapp & Merilä, 2018). Evolutionary response to climate‐mediated selection is possible only if a population possesses heritable variation in the traits under natural selection (Lynch & Walsh, 1998). Both the force of natural selection (e.g. Vatka et al., 2021) and trait heritabilities (Hoffmann & Merilä, 1999; De Neve et al., 2004; Charmantier & Garant, 2005; Rowiński & Rogell, 2017) can change either independently of each other or jointly (Merilä, 1997; Ramakers et al., 2018; Wilson et al., 2006; Wood & Brodie, 2016) with changing environmental conditions. Hence, to predict evolutionary responses to changing environmental conditions, it becomes important to understand how changing conditions influence trait heritabilities and underlying variance components. However, demonstrations of climate‐change‐associated changes in quantitative genetic parameters are rare (but see Gunay et al., 2011).

Body size is an important life‐history trait (Messina & Fox, 2001) that is heritable in most species studied (Postma, 2014). There has been a lot of recent interest in documenting and understanding how and why the body size of many organisms is impacted by climate change (e.g. Daufresne et al., 2009; Isaac, 2009; Merilä & Lv, 2025; Sheridan & Bickford, 2011). While climate‐driven or associated changes in mean body size have been documented in many studies (e.g. Merilä & Lv, 2025; Sheridan & Bickford, 2011; Teplitsky et al., 2008), such changes are not universal (Radchuk et al., 2019), and it has proven challenging to disentangle the underlying genetic versus environmental causes of these trends (Merilä & Hendry, 2014, but see Bonnet et al., 2017).

Body size and its heritability are affected by environmental conditions such as food availability (e.g. Gebhardt‐Henrich & van Noordwijk, 1991; Larsson, 1993; Larsson et al., 1997; Merilä, 1997; Rowiński & Rogell, 2017). Climate warming can influence predators' food availability through changes at different levels of a food chain: in primary production (Boisvenue & Running, 2006), northwards shifting ranges of prey species (Hällfors et al., 2021; Pöyry et al., 2009) and/or changes in temporal match between the timing of the prey availability peak and the timing of the predators' breeding periods (e.g. Vatka et al., 2011, 2014; Visser et al., 2006). It is thus conceivable that climate‐change‐driven trends in food availability influence also the body size of the predator species, its heritability and evolvability.

We used long‐term data (Vatka et al., 2025) from two individually marked woodland passerines, the Willow Tit (*Poecile montanus*) and the Great Tit (*Parus major*), to study how variation in caterpillar food availability affected nestling's morphological traits, their heritabilities and evolvabilities. When focussing on the nestling stage (instead of size measures from adults), we expect to capture the population's full evolutionary potential regarding body size traits, as when reaching the adult stage some of the underlying additive genetic variance has possibly already been weeded out by selection. In the Willow Tit population, synchrony with the food peak has improved with climate warming (Vatka et al., 2011), whereas no detectable change in synchrony has been observed for the Great Tit (Vatka et al., 2014). We hypothesized that increasing food availability would be associated with increased nestling body size in both populations and that low food availability would reduce heritabilities (and evolvabilities) of body size traits. The latter would be expected if selection weeds out fast‐growing genotypes starving under low food availability reducing additive genetic variance and increasing the environmental component of variance. Conversely, high trait heritabilities (and evolvabilities) could be expected under high food availability if there is less mortality and a wider array of genotypes survive to fledge. Under this scenario, the expectation is an increase in additive genetic and a decrease in the environmental component of variance. While these predictions align with the results of earlier experimental work, which shows that reduced access to food can reduce trait heritabilities (Merilä, 1997), there are many other possible outcomes (e.g. Hoffmann & Merilä, 1999; Rowiński & Rogell, 2017) and we come back to these in the discussion.

## 2 MATERIALS AND METHODS

### 2.1 Study species

The study species, the Willow Tit and the Great Tit, are passerines that inhabit woodlands in Europe and Asia. Our study areas in boreal forests in Oulu (N 65°05′, E 25°33′), Northern Finland (Pakanen et al., 2016), are occupied by ca. 76 (range: 13–162) pairs of Willow Tits and ca. 80 (range: 11–138) pairs of Great Tits. The populations have been intensively monitored since 1975 and 1969, respectively. The two study species differ in their distribution histories, the Willow Tit being a native species in Northern Europe while the Great Tit has become abundant in Northern Finland only during the first half of the 20th century (Haartman et al., 1967). Great Tits lay large clutches but have high nestling mortality in the north (Rytkönen & Orell, 2001), whereas Willow Tits seem to follow a clutch adjustment tactic, showing less nestling mortality (Orell & Ojanen, 1983a).

### 2.2 Data collection on morphological traits

Data on morphological measures of Willow Tit and Great Tit nestlings were collected with standard methods (Orell, 1983; Orell & Ojanen, 1983a) during years 1996–2020 (for which food availability data were also available). All found nests were visited regularly to determine the timing of breeding and other breeding parameters. For each nest, the onset of egg laying was determined by counting back from the date of the first observation of eggs in a nest, under the assumption that one egg was laid a day. The clutch size is the total number of eggs in a completed clutch (i.e. the number of eggs in an incubated nest). The date of hatching was defined by observing the habitus of recently hatched chicks in nests visited repeatedly from the expected hatching day (13–14 days after the laying date of the last egg of the clutch; Orell & Ojanen, 1983a) until the observed hatching. Nestlings were measured at the age of 2 weeks (13 or 14 days old for the Willow Tit and the Great Tit, respectively; 0 = hatching date of the oldest young). Measuring and marking of nestlings was done at this age, as the nestlings are already close to the fledging size, but still young enough so that the disturbance caused by handling would not provoke the young to leave their nests too early—fledging as underdeveloped would be detrimental for the young. Wing length and tail length were measured using a ruler to the nearest mm (Orell, 1983). Tarsus length was measured using a vernier calliper to the nearest 0.01 mm (Svensson, 1992), and body mass with either a Pesola spring balance or digital scales to the nearest 0.1 g. We note that measurements are not strictly speaking adult traits, but the tarsus length has reached its final size at the age nestlings were measured, and hence, is a proxy of adult body size. Wing, tail and tarsus lengths are prone to measurement error as different measurers hold the birds differently while taking the measures. This between‐measurers variation was accounted for in the analyses (see below). The Willow Tit data consisted of measurements of 11,568 nestlings from 1730 broods. For the Great Tit, the sample size was 7629 nestlings from 1140 broods. For detailed sample sizes for each size trait (excluding missing values), please see Tables S1 and S2.

### 2.3 Pedigree data

Both the nestlings and adult birds have been marked with aluminium rings with unique codes. For marking of adults, individual colour codes with plastic colour rings are also used; this aids their later identification (observations with binoculars) without a need to catch the parent birds again. Individual marking enables the identification of recruits and the building of pedigrees. A pruned Willow Tit pedigree reached 13 generations in maximum depth, consisting of approximately 13,000 individuals. Of fledged Willow Tit offspring, approximately 5% recruited to the study population as breeding adults. For the Great Tit, the pruned pedigree was 10 generations in maximum depth and included approximately 9000 individuals. The recruitment rate for the Great Tit is approximately 4%. For detailed numbers of individuals in pruned pedigrees for each analysis, please see Tables S1 and S2.

All research presented in the article was conducted in accordance with all applicable laws and rules governing the ethical treatment of animals as set forth by the Finnish Government. Visiting the nests, catching the birds and conducting research in the Natura 2000 nature reserve was permitted by the Centre for Economic Development, Transport and the Environment (permit numbers VARELY/1008/2018 and POPELY/754/2018). Ringing of birds was done under a ringing licence provided by the Finnish Natural History Museum, University of Helsinki. This study did not require approval from an animal ethics committee.

### 2.4 Food availability data and temporal trends

During the nestling period, Willow Tit and Great Tit parents feed their offspring mostly with caterpillars when available (e.g. Perrins, 1991; Rytkönen et al., 1996; Rytkönen & Orell, 2001). Caterpillar food availability during nestling periods was quantified with the frass‐fall method (Zandt, 1994) as described in Rytkönen and Orell (2001) for the years 1996–2020. Biomasses of caterpillars (g/m^2^) living in canopies of birch (*Betula* spp.) were estimated in weekly periods. We assumed that the weekly caterpillar biomass estimate reflects daily caterpillar abundances during the frass‐fall collection period. We then calculated brood‐specific average caterpillar food availabilities per day during nestling periods (from the hatching date until the date of nestlings' measurement). Thus, broods that hatched on the same day each year have the same food availability estimates. The unit for food availability is g/m^2^/day.

We used linear mixed effect models with the function ‘lmer’ in the library ‘lme4’ (Bates et al., 2015) in R 4.3.1 (R Core Team, 2023) to test for temporal changes in brood‐specific food availabilities (*F*) over the study period of 25 years. The model had the following structure:

(1) $$F_{\mathrm{ij}}=\alpha +\beta \text{Year}+{\text{female}}_{i}+{\text{year}}_{j}+e_{\mathrm{ij}}$$

where *α* is the intercept and *β* is the regression coefficient estimate for the fixed effect of year (treated as a continuous variable). Female ID and year were used as block random factors to account for repeated measures from the same females and years. We also tested for adding male ID as a block random factor to the model, but that resulted in either a singular fit or non‐convergence and thus male ID was excluded from the model. *e* is the error term.

To define two‐tailed *p*‐values for the regression coefficients (function ‘pt’ in library ‘stats’), we defined degrees of freedom (df) as

(2) $$\begin{array}{c} \mathrm{df}=N\left(\text{observations}\right)-N\left(\text{fixed effect estimates}\right)\\ \;-N\left(\text{random effect levels}\right) \end{array}$$

### 2.5 Effects of food availability on nestlings' body size traits and brood size

We built linear mixed effect models with the function ‘lmer’ in the library ‘lme4’ to test whether body size measures (individual values of wing length, tail length or tarsus length; *y*) of nestling birds were influenced by food availability (*F*). We used the following model structure as a starting point:

(3) $$\begin{array}{c} y_{\text{hijklm}}=\alpha +\mathrm{\beta F}+{\text{year}}_{j}+{\text{brood}}_{k\left(i\right)}+{\text{female}}_{i}+{\text{male}}_{l}\\ \;+{\text{measurer}}_{m}+e_{\text{hijklm}} \end{array}$$

Year, brood ID (nested within female ID), female ID and male ID were used as block random variables to deal with interdependencies of observations. The measurer term was used to account for variance due to slight differences in how people measure different traits. To test the effect of food availability on nestlings' body mass, we used the same model structure (Equation 3), but excluded the measurer from the random effects, as body mass is measured with scales that are not affected by the measurer. Male ID was excluded from models describing Great Tit tarsus length and body mass, and female ID from analyses on the Willow Tit tail length, due to convergence issues with the full model structures.

To indirectly test our assumption that food availability would affect mortality rates of nestlings and thus the array of surviving genotypes, we tested whether food availability affected the brood size (number of nestlings at the age of 2 weeks, the time of measurements) by using generalized linear mixed effect models with Poisson error structures with function ‘glmer’ (library ‘lme4’), using log link function:

(4) $$\log\left({\text{brood size}}_{\mathrm{kj}}\right)=\alpha +\mathrm{\beta F}+{\text{year}}_{j}+e_{\mathrm{kj}}$$

Year was used as a block random variable (adding female or male IDs as block random variables would result in singular fits).

### 2.6 Temporal trends in nestling birds' body size traits

We tested temporal trends in body size measures with linear mixed effect models by regressing individual values of wing length, tail length, tarsus length and body mass against year. We started with the model structure given in Equation (5):

(5) $$y_{\text{hiklm}}=\alpha +\beta \text{Year}+{\text{brood}}_{k\left(i\right)}+{\text{female}}_{i}+{\text{male}}_{l}+{\text{measurer}}_{m}+e_{\text{hiklm}}$$

For the Willow Tit tail length, this model resulted in a singular fit and thus, female ID was excluded. For the body mass, the model structure of Equation (5) was followed but the measurer term was excluded, and in the case of the Great Tit, male ID was also omitted due to a singular fit with the full model structure.

### 2.7 Random regression animal models

We estimated how additive genetic variances and heritabilities of body size traits of nestling birds vary with food availabilities (considered as a continuous variable). This was done by building random regression animal models that utilize information of relatedness values given by the pedigrees and of morphological measures in varying food availabilities. Food availability values were standardized by subtracting the mean and dividing by standard deviation to ease the interpretation of the resulting variance components. The function ‘MCMCglmm’ (library ‘MCMCglmm’; Hadfield, 2010) was used to fit models in R. A body size measure (viz. wing length, tail length or tarsus length; *y*) of the individual *h* in year *j* in food availability *f* was modelled as:

(6) $$y_{\text{fhjkm}}=\alpha +\beta F_{f}+{\text{year}}_{j}+{\text{measurer}}_{m}+{\text{brood}}_{k}+A_{h}+B_{h}F_{f}+e_{\text{fhjkm}},$$

where *α* is the intercept and *β* is a regression coefficient for a fixed effect food availability ($F_{f}$). Year (${\text{year}}_{j}$) and measurer (${\text{measurer}}_{m}$) were used as block random factors with estimated variances of ${\text{year}}_{j}\sim N\left(0,\sigma_{\text{year}}^{2}\right)$ and ${\text{measurer}}_{m}\sim N\left(0,\sigma_{\text{measurer}}^{2}\right)$. Brood ID (${\text{brood}}_{k}$) was included in the model to capture permanent environmental effects with estimated variance of ${\text{brood}}_{k}\sim N\left(0,\sigma_{\text{brood}}^{2}\right)$. $A_{h}$ and $B_{h}$ are random intercepts and slopes of the additive genetic component. Additive genetic variance was estimated using a 2 × 2 variance–covariance matrix:

(7) $$G=\left[\begin{array}{cc} \sigma_{A}^{2} & \sigma_{A,B}\\ \sigma_{A,B} & \sigma_{B}^{2} \end{array}\right]$$

$e_{\text{fhjkm}}$ (Equation 6) is the residual term. Possible heteroscedasticity of residual variance across varying levels of food availability was considered by estimating the residual variance for each equal‐interval group *l* of food availabilities as $e_{\text{fhjkm}}\sim N\left(0,\sigma_{e,l}^{2}\right)$ (Ramakers et al., 2020). The number of groups *n* = 10 was used.

For body mass, a similar model structure (Equation 6) was used with the exception that the measurer term was excluded from the model.

As a default, we used a wide normal distribution as a prior for fixed factors. For the residual variance, we used an inverse‐Wishart prior with *V* = diag(*n*) and nu = 0.002. For other variance components, parameter‐expanded priors (*V* = diag(*x*), nu = *x*, alpha.mu = 0, alpha.*V* = diag(*x*) × 1000) were used. For each model, a total of 10,100,000 MCMC iterations were run, including a burn‐in period of 100,000 iterations. The remaining 10,000,000 iterations were sampled with a thinning interval of 10,000, leading to sample sizes of 1000 saved iterations.

We estimated additive genetic variances (*V*_*A*_) and heritabilities (*h* ^2^) for each documented food availability value, for each of the saved 1000 iterations. This created distributions of estimated values, of which median values are reported with highest posterior density intervals (HPD intervals) as confidence limits (appendix of Hadfield et al., 2010; Vatka et al., 2021). *V*_*A*_ for each food availability value $F_{f}$ were derived using the **G** matrix as

(8) $$V_{A_{f}}=\sigma_{A}^{2}+2\sigma_{A,B}F_{f}+\sigma_{B}^{2}F_{f}^{2}$$

Food availability dependent heritability was calculated as

(9) $$h_{f}^{2}=\frac{V_{A_{f}}}{V_{A_{f}}+\sigma_{\text{year}}^{2}+\sigma_{\text{brood}}^{2}+\sigma_{e,l}^{2}},$$

The effect of food availability on *V*_*A*_ or *h* ^2^ was considered statistically significant, if the minimum value of the upper HPD interval limit values was smaller than the maximum value of the lower HPD interval limit values.

### 2.8 Evolvabilities of nestlings' body size traits in varying food availabilities

We estimated evolvabilities of nestlings' body size traits for varying food availability values as mean scaled additive genetic variances (*I*_*A*_; Hansen et al., 2024) using the formula

(10) $${I_{A}}_{f}=\frac{V_{A_{f}}}{{\bar{x}}_{f}^{2}}$$

where ${\bar{x}}_{f}$ is the trait mean in a food availability *f*. To calculate these mean trait values, we divided food availabilities into 50 equal‐interval groups and calculated mean values (and standard errors) of trait values for each food availability group. The uncertainty of the evolvability estimates were calculated as 95% confidence intervals, considering uncertainties of both *V*_*A*_ and $\bar{x}$:

(11) $$95\%{\mathrm{CI}}_{f}=I_{A_{f}}\pm 1.96\times \sqrt{{\frac{\mathrm{SE}\left({V_{A}}_{f}\right)}{{V_{A}}_{f}}}^{2}+{\left(2\frac{\mathrm{SE}\left({\bar{x}}_{f}\right)}{{\bar{x}}_{f}}\right)}^{2}}\times {I_{A}}_{f}$$

For calculations of mean scaled environmental variances (CV_E_) for varying food availability values, please see Supporting Information S1.

## 3 RESULTS

### 3.1 Temporal change in food availability

**FIGURE 1:**
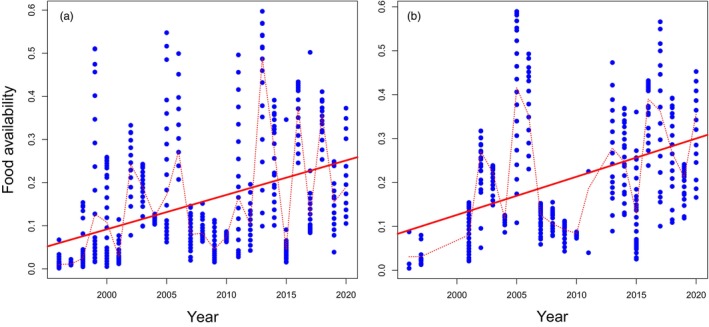
Temporal variation and overall increases in brood‐specific values of average caterpillar food availabilities (g/m^2^/day, blue dots) during the nestling phase in the Willow tit (a) and the Great Tit (b) in Oulu, Finland. Solid red lines present temporal trends (regression lines), whereas dotted red lines connect annual average values and show year‐to‐year variation.

When inspecting changes in caterpillar food availability for nestlings, a positive temporal trend over the 25‐year‐long study period was found for both the Willow Tit (*b* = 0.008, SE = 0.003, *p* = 0.009; Figure 1a) and the Great Tit (*b* = 0.009, SE = 0.003, *p* = 0.010; Figure 1b). In general, food is now more abundant during the nestling period than before, although there is considerable within‐year variation in brood‐specific food availabilities as well as year‐to‐year variation in annual mean values (Figure 1). However, this result must be taken with caution, as the normality assumptions for model residuals (inspected with QQ‐plots) were not fully met.

### 3.2 Covariation among food availability, body size traits and brood size

A majority of body size traits covaried with variation in food availability in both the Willow Tit and the Great Tit, trait values being larger with higher food abundances (Table 1). The only exception was the Willow Tit tarsus length that did not show a statistically significant association with the food availability (Table 1).

**TABLE 1:** Effects of changing food availabilities (g/m^2^/day) on body size traits based on linear mixed effect models with food availability as an explanatory variable.

Response variable	Willow Tit				Great Tit			
	*b*	SE	*t*	*p*	*b*	SE	*t*	*p*
Wing length	4.636	0.940	4.934	<0.001	10.074	1.691	5.956	<0.001
Tail length	4.868	0.913	5.333	<0.001	10.199	1.678	6.080	<0.001
Tarsus length	0.220	0.146	1.506	0.132	0.791	0.261	3.031	0.002
Body mass	0.490	0.235	2.085	0.037	2.331	0.664	3.512	<0.001

*Note*: Please see Section 2 for full model structures. Units for wing, tail and tarsus lengths are mm, for body mass g.

The brood size of Willow Tits was not associated with food availability (*b* = −0.009, SE = 0.107, *p* = 0.936). By contrast, the brood size of the Great Tit was positively associated with food availability, the numbers of measured nestlings being higher in high food abundances (*b* = 0.430, SE = 0.142, *p* = 0.003).

### 3.3 Temporal trends in body size traits

The body mass of Willow and Great Tit nestlings has on average increased over time during the 25‐year study period (Table 2): Willow tit nestlings were approximately 0.3 g and Great Tit nestlings appr. 0.5 g heavier at the end of the study period than at its beginning. Wing, tail or tarsus lengths did not show statistically significant temporal trends in either species (Table 2).

**TABLE 2:** Temporal changes in body size traits based on linear mixed effect models with year as an explanatory variable.

Response variable	Willow Tit				Great Tit			
	*b*	SE	*t*	*p*	*b*	SE	*t*	*p*
Wing length	−0.021	0.013	−1.565	0.118	0.033	0.043	0.771	0.441
Tail length	−0.010	0.013	−0.786	0.432	0.015	0.042	0.363	0.717
Tarsus length	0.004	0.002	1.623	0.105	0.004	0.007	0.552	0.581
Body mass	0.012	0.003	4.043	<0.001	0.021	0.010	2.137	0.033

*Note*: Please see Section 2 for full model structures. Units for wing, tail and tarsus lengths are mm, for body mass g.

### 3.4 Food availability and heritabilities

**FIGURE 2:**
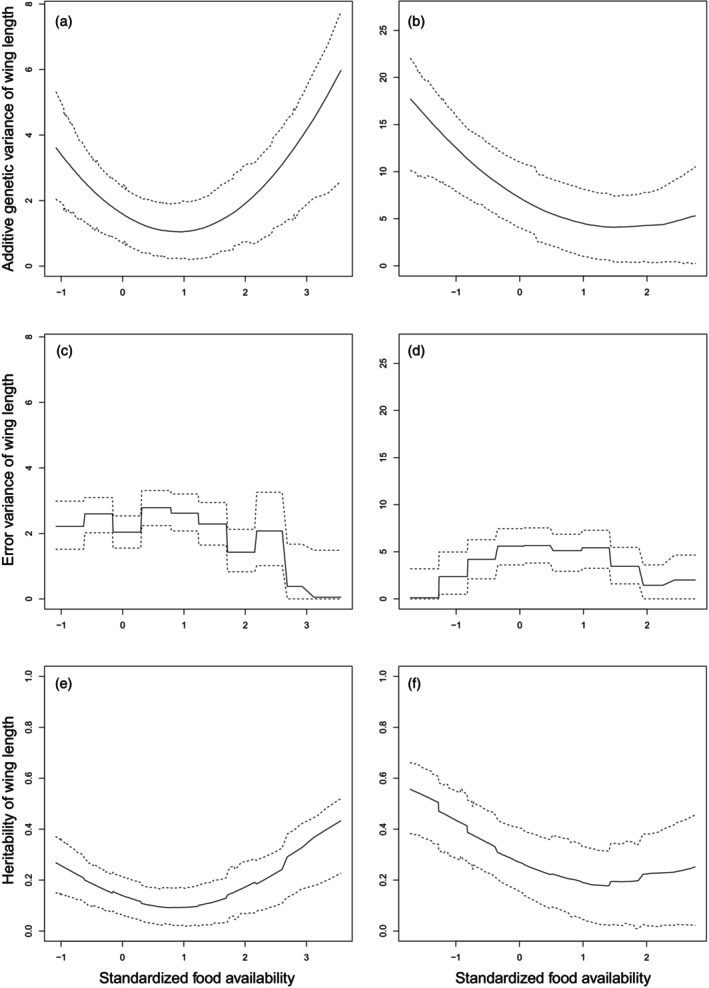
Additive genetic variance (a, b), error variance (c, d) and heritability estimates (e, f) of wing length in nestling Willow (a, c, e) and Great Tits (b, d, f) in different caterpillar food availabilities. Food availability has been standardized by subtracting the mean and dividing by standard deviation. Solid lines are median values of estimates for 1000 iterations, and dotted lines present HPD intervals for the distributions of estimated values (please see Section 2 for details).

According to the random regression animal models, food availability influenced additive genetic variance (*V*_*A*_) and heritability of wing length in both study species (Tables 3 and 4; Figure 2). For the Willow Tit, the additive genetic variance was lowest at moderate food availability values and increased for both small and large values (Figure 2a). For the Great Tit the additive genetic variance decreased with increasing food availability (Figure 2b). Residual variance estimates varied across food availabilities but did not show clear patterns for decreasing with increasing food availabilities (Figure 2c,d).

**TABLE 3:** Model estimates from the random regression animal model for the wing length (mm) of the Willow Tit.

Parameter	Posterior mode	95% HPD interval		Effective sample size
Fixed effects				
Intercept	39.157	38.153	39.981	1000.0
Food availability	0.626	0.361	0.851	1232.0
Random effects				
*V*_*Y*_ $\sigma_{\text{year}}^{2}$	1.566	0.877	3.110	1000.0
*V*_PE_ $\sigma_{\text{brood}}^{2}$	5.985	5.403	6.526	1000.0
*V*_*M*_ $\sigma_{\text{measurer}}^{2}$	1.668	0.340	3.794	1253.0
*V*_*A*_ $\sigma_{A}^{2}$	1.255	0.708	2.417	1000.0
$\sigma_{A,B}$	−0.594	−1.019	−0.149	1000.0
$\sigma_{B}^{2}$	0.651	0.304	0.976	1332.0
*V*_*R*_ $\sigma_{e,\left(-1.090--0.621\right)}^{2}$	2.229	1.518	2.986	1000.0
$\sigma_{e,\left(-0.621--0.157\right)}^{2}$	2.536	2.020	3.099	1000.0
$\sigma_{e,\left(-0.157-0.308\right)}^{2}$	2.060	1.552	2.535	1000.0
$\sigma_{e,\left(0.308-0.772\right)}^{2}$	2.881	2.240	3.307	843.3
$\sigma_{e,\left(0.772-1.240\right)}^{2}$	2.661	2.075	3.205	1007.9
$\sigma_{e,\left(1.240-1.700\right)}^{2}$	2.355	1.645	2.941	901.7
$\sigma_{e,\left(1.700-2.170\right)}^{2}$	1.349	0.833	2.127	875.4
$\sigma_{e,\left(2.170-2.630\right)}^{2}$	1.916	1.020	3.255	1000.0
$\sigma_{e,\left(2.630-3.090\right)}^{2}$	0.009	0.000	1.671	990.9
$\sigma_{e,\left(3.090-3.560\right)}^{2}$	0.008	0.000	1.485	1000.0

*Note*: $\sigma_{A}^{2}$ = variance of random intercepts; $\sigma_{B}^{2}$ = variance of random slopes; $\sigma_{A,B}$ = covariance of random intercepts and slopes; $\sigma_{e}^{2}$ = error variances in different food availability blocks. Food availability has been standardized by subtracting the mean and dividing by standard deviation.

**TABLE 4:** Model estimates from the random regression animal model for the wing length (mm) of the Great Tit.

Parameter	Posterior mode	95% HPD interval		Effective sample size
Fixed effects				
Intercept	47.221	45.737	48.093	1000.0
Food availability	1.454	0.935	1.796	1000.0
Random effects				
*V*_*Y*_ $\sigma_{\text{year}}^{2}$	2.553	1.089	6.725	905.2
*V*_PE_ $\sigma_{\text{brood}}^{2}$	9.691	8.798	11.895	1000.0
*V*_*M*_ $\sigma_{\text{measurer}}^{2}$	1.968	0.242	5.818	1000.0
*V*_*A*_ $\sigma_{A}^{2}$	7.308	4.070	11.020	784.7
$\sigma_{A,B}$	−1.839	−2.916	−0.866	688.9
$\sigma_{B}^{2}$	1.070	0.249	2.152	1120.3
*V*_*R*_ $\sigma_{e,\left(-1.720--1.270\right)}^{2}$	0.012	0.000	3.187	890.4
$\sigma_{e,\left(-1.270--0.821\right)}^{2}$	2.059	0.492	4.975	869.8
$\sigma_{e,\left(-0.821--0.373\right)}^{2}$	4.737	2.128	6.284	875.0
$\sigma_{e,\left(-0.373-0.0742\right)}^{2}$	5.361	3.609	7.449	762.6
$\sigma_{e,\left(0.0742-0.522\right)}^{2}$	5.707	3.805	7.539	700.7
$\sigma_{e,\left(0.522-0.970\right)}^{2}$	5.372	2.932	6.849	658.3
$\sigma_{e,\left(0.970-1.420\right)}^{2}$	5.853	3.237	7.283	453.9
$\sigma_{e,\left(1.420-1.870\right)}^{2}$	4.368	1.598	5.474	440.3
$\sigma_{e,\left(1.870-2.310\right)}^{2}$	0.019	0.000	3.611	318.6
$\sigma_{e,\left(2.310-2.760\right)}^{2}$	0.024	0.001	4.651	347.8

*Note*: $\sigma_{A}^{2}$ = variance of random intercepts; $\sigma_{B}^{2}$ = variance of random slopes; $\sigma_{A,B}$ = covariance of random intercepts and slopes; $\sigma_{e}^{2}$ = error variances in different food availability blocks. Food availability has been standardized by subtracting the mean and dividing by standard deviation.

The random regression animal models did not recover statistical support for effects of changing food availabilities in additive genetic variance or heritability in tail length (Tables S3 and S4; Figure S1), tarsus length (Tables S5 and S6; Figure S2) or body mass (Tables S7 and S8; Figure S3) in neither the Willow Tit nor the Great Tit. Heritability estimates for tarsus length were somewhat higher for the Willow Tit (*h* ^2^≈0.31) than for the Great Tit (*h* ^2^≈0.18; Figure S2). Regarding other body size traits, heritability estimates were approximately of the same scale for the two species: for tail length *h* ^2^≈0.18 and *h* ^2^≈0.25 (Figure S1), and for body mass *h* ^2^≈0.31 and *h* ^2^≈0.27 (Figure S3) for the Willow Tit and the Great Tit, respectively.

### 3.5 Food availability and evolvabilities

**FIGURE 3:**
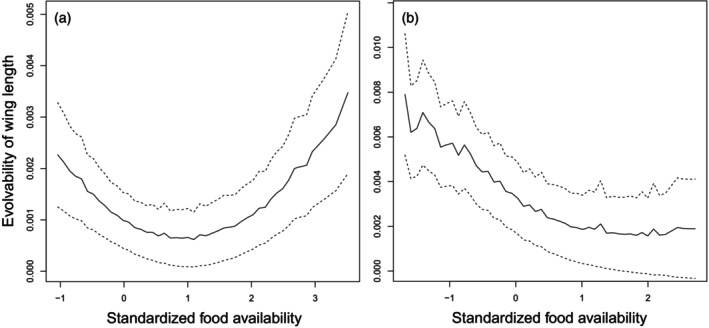
Evolvabilities of wing length in nestling Willow (a) and Great Tits (b) in different caterpillar food availabilities. Food availability has been standardized by subtracting the mean and dividing by standard deviation. Solid lines are mean values of estimates, and dotted lines present 95% confidence intervals (please see Section 2 for details).

Evolvabilities of wing length in nestling Willow and Great Tits were influenced by caterpillar food availabilities (Figure 3). The patterns are similar to what was observed for the additive genetic variances and heritabilities: for the Willow Tit, evolvability of wing length was lowest at moderate food availability values and higher for both small and large values (Figure 3a), and for the Great Tit evolvability decreased for higher food availabilities (Figure 3b). Evolvability values of other nestlings' body size traits (tail length, tarsus length and body mass) did not show statistically significant associations with food availability in either study species (Figure S4).

## 4 DISCUSSION

In this study, we found evidence for an (apparently climate warming associated) increase in food availability for nestlings of two passerine bird species, as well as concomitant trends in mean values of nestling body mass. Most interestingly, the heritability and evolvability of wing length responded to increasing levels of caterpillar food availability in both the Willow Tit and the Great Tit, albeit in different ways. No detectable effect of varying food availability on heritabilities or evolvabilities of other traits was found.

The observed average increase (i.e. a positive temporal trend) in caterpillar food availability over time during the nestling phase may arise from various mechanisms. The timing of the larval period of the prey and the timing of the nestling period of the predator may or may not match. According to previous studies in this study system, there is year‐to‐year variation in the synchrony between the caterpillar availability and nestling periods in the Willow Tit (Vatka et al., 2011) and the Great Tit (Vatka et al., 2014). In the Willow Tit, the synchrony has improved with the caterpillar peaks shifting earlier in warmer springs (Vatka et al., 2011). Also, the overall abundance of caterpillars varies from year to year, owing to variations in population sizes of the prey, but possibly also due to northward shifts of distributions of more southern prey species (Hällfors et al., 2021; Pöyry et al., 2009). These factors, the synchrony and the abundance, likely contribute together to the observed increase in food availability. Hence, in contrast to many other bird systems where the climate change‐driven environmental trends have had predominantly negative effects (e.g. Teplitsky et al., 2008; review in: Sheridan & Bickford, 2011), the results of this study suggest generally positive effects on these boreal populations of hole‐nesting passerines. Our results stand in stark contrast with those of Husby et al. (2011) who found a declining body mass trend in three Dutch populations of Great Tits. Similar body mass declines are also reported from two British populations of Great Tits (Cresswell et al., 2009; Yom‐Tov et al., 2006). Hence, our results of positive temporal trends in body mass highlight that the effects of climate change on organismal populations can be geographically heterogeneous.

We originally expected that increasing food availability would translate to increased heritability of nestling size as more surviving nestlings can be expected incorporate broader range of growth genotypes and thereby lead to higher levels of expressed additive genetic variation (*V*_*A*_) as observed in earlier studies of birds (reviewed in Hoffmann & Merilä, 1999; Merilä & Sheldon, 2001). When testing our premise, we found that higher food availabilities increased brood sizes of Great Tits, but not of Willow Tits. In general, Great Tits suffer from higher nestling mortality than Willow Tits which seem to be better adapted to life in boreal forests at high latitudes (Orell & Ojanen, 1983a, 1983b). Yet, the number of fledged young correlated positively with caterpillar food availability in both study species (Vatka et al., 2014). One potential explanation for these observations is that all nestlings with different genotypes can grow fast under favourable conditions, but low food availability amplifies individual differences in their growth potential. This should result in negative covariances between the intercept and slope of the additive genetic component ($\sigma_{A,B}$), as was found for the wing length in both Willow Tits (Table 3) and Great Tits (Table 4), as well as for the body mass of the Willow Tit nestlings (Table S7). Parents may distribute food differently among the nestlings depending on food availability (Zhu et al., 2023)—preferential allocation of food for (genetically) fastest growing nestlings under food scarcity could amplify genotypic and phenotypic variance among nestlings, as well as increase environmental variance.

Our results from random regression animal models suggest that food availability affected expression of additive genetic variation in the wing length of both the Willow and the Great Tit. However, for the Willow Tit the response curve was U‐shaped, indicating that both very low and high food availabilities increased expression of additive genetic variance. In contrast, the lower food availabilities were associated with the highest additive genetic variances in the case of Great Tits. Both these results collide with our original hypothesis. One possible explanation for these findings is technical: as variances are always positive, and as Formula (7) used to calculate $V_{A_{k}}$ has a form of an equation of the second degree, $V_{A_{k}}$ will always have a U‐shaped curve. This should hold true also in case of the Great Tit, but they might never experience truly high food availability in our northern study area. For example, Rytkönen and Krams (2003) reported manifold caterpillar food availabilities during nestling phase in Latvia compared to our study area in Northern Finland.

We can only speculate potential ecological explanations for U‐shaped curves of expressed genetic variation (*V*_*A*_), heritability and evolvability of wing length. As mentioned above, it could be that very low food availabilities amplify individuals' genetic differences in growth potential. Under mediocre food availabilities some genotypic differences might be overridden by environmental effects. The increased expression of additive genetic variance under high food availability discovered in the Willow Tit could perhaps be explained with higher between‐brood variance: if the most favourable conditions enable also genetically slowly growing individuals to produce offspring (that inherit low growth rates), it might translate to higher levels of expressed genetic variation at a population level.

Our approach for animal models allowed the residual variances to vary across food availabilities—other parts of environmental variation (*V*_PE_ and *V*_year_) were estimated as constants. We hypothesized that environmental variation would decrease with increasing food availability. However, this type of pattern was not found, at least unambiguously.

The other studied morphological traits (viz. tail length, tarsus length and body mass) were also heritable, but their heritabilities and evolvabilities were constant over different food availabilities. Thus, the findings regarding the wing length cannot be generalized to other body size traits. In matter of fact, the results regarding the best proxy of body size (tarsus length) suggest that the evolutionary potential of body size is not sensitive to variation in food availability. The results are consistent with similar experimental evidence from the Blue Tit (*Cyanistes caeruleus*; Merilä & Fry, 1998), as well as from results of a meta‐analysis of morphological traits in animals (Rowiński & Rogell, 2017).

In this study, we addressed body size traits of offspring (instead of adults). The offspring express genetic variation welling up from sexual reproduction that creates new recombinants and thus genotypic and phenotypic variation in a population. Some of these variations can be lost due to strong selection that takes place before adulthood. When addressing offspring size, our intention was not to measure the reproductive investments or the fitness of the parents (though offspring size is often used as a metric for adults' fitness), but to address the phenotypic and genetic variation of body size traits in a population at a life stage when they are at their amplest.

While tarsus length had reached its final size at the time the nestlings were measured, wing and tail lengths were still growing (Orell, 1983). Therefore, the heritabilities for these traits likely reflect genetic differences in the growth rate of individuals, rather than that of wing or tail length of full‐grown individuals sensu stricto. In this perspective, the fact that additive genetic variance in growth rate changed with food availability in both species suggests that the evolutionary potential of growth rates appears to be sensitive to food availability. For instance, in the case of the Great Tit, expected response to selection on growth rate would be faster in low than in high food availability. Selection pressures on growth rate may arise as more slowly growing individuals can stay in the nest for a longer period than the fast‐growing ones (Orell, 1983), which exposes nestlings to increased risk of nest predation (Martin et al., 2018). Yet, fledging at a younger age but with shorter wings could compromise survival after fledging due to reduced ability to escape predators (Martin et al., 2018) such as the Sparrowhawk (*Accipiter nisus*).

The evolution of different traits can be intertwined. For example, if individuals that hatch early in the breeding season experience higher food availabilities than later hatching individuals, and if food availability affects heritability of the growth rate as suggested by the results of this study, selection acting on the timing of breeding (i.e., early hatched individuals are more likely to recruit to the population; Vatka et al., 2021) will likely affect the evolutionary potential in the growth rate as well. Similarly, due to size dimorphism between sexes (e.g. Björklund & Lindén, 1993), parent birds may need to allocate food resources differently between female and male offspring when food is scarce. If one sex is preferred to another, it might negatively affect the survival of the less cared for young of a certain sex, leading to a change in the offspring sex ratio—another life history trait that may be connected to the relationship between food availability and growth rate.

An environmental variable, such as food availability, can thus affect the evolutionary potential of a trait. As there is distinct year‐to‐year variation in this environmental variable (Figure 1), the heritability of the given trait must also vary from year to year. However, as there are also varying levels of within‐year variation in food availabilities (Figure 1), each cohort of nestlings may include individuals with differing levels of expression of their genotype (i.e., phenotypes of some individuals are closer to their breeding values than those of others). Hence, the amount of variation in the level of genetic expression between individuals must vary between years. How this affects the evolutionary potential of populations remains to be explored.

## 5 CONCLUSIONS

We found a temporal trend for increasing food availability and body mass in two hole‐nesting passerines, but no evidence that the additive genetic variance in body size would differ across food availabilities. However, additive genetic variance in the nestling wing length—a proxy of growth rate—showed contrasting dependency on food availability, indicating that the evolutionary potential in growth rates varies as a function of food availability. Further studies are needed to understand why the expression of additive genetic variance in response to food availability differed between the two species and what causes the U‐shape in curves of expressed genetic variation and heritability of wing length.

## AUTHOR CONTRIBUTIONS

Emma Vatka is responsible for the conception and design of the article, as well as for conducting the analysis. Markku Orell, Emma Vatka and Seppo Rytkönen collected the data. Emma Vatka, Juha Merilä and Markku Orell drafted the article; Seppo Rytkönen revised it. All authors gave their final approval for the article publication.

## CONFLICT OF INTEREST STATEMENT

The authors declare no conflicts of interest.

## Supporting information


**Table S1.** Sample sizes for the Willow Tit, and numbers of individuals in pruned pedigrees.
**Table S2.** Sample sizes for the Great Tit, and numbers of individuals in pruned pedigrees.
**Table S3.** Model estimates from the random regression animal model for the tail length of the Willow Tit.
**Table S4.** Model estimates from the random regression animal model for the tail length of the Great Tit.
**Figure S1.** Additive genetic variance (A, B), error variance (C, D) and heritability estimates (E, F) of tail length in nestling Willow (A, C, E) and Great Tits (B, D, F) in different caterpillar food availabilities.
**Table S5.** Model estimates from the random regression animal model for the tarsus length of the Willow Tit.
**Table S6.** Model estimates from the random regression animal model for the tarsus length of the Great Tit.
**Figure S2.** Additive genetic variance (A, B), error variance (C, D) and heritability estimates (E, F) of tarsus length in nestling Willow (A, C, E) and Great Tits (B, D, F) in different caterpillar food availabilities.
**Table S7.** Model estimates from the random regression animal model for the body mass of the Willow Tit.
**Table S8.** Model estimates from the random regression animal model for the body mass of the Great Tit.
**Figure S3.** Additive genetic variance (A, B), error variance (C, D) and heritability estimates (E, F) of body mass in nestling Willow (A, C, E) and Great Tits (B, D, F) in different caterpillar food availabilities.
**Figure S4.** Evolvabilities of tail length (A, B), tarsus length (C, D) and body mass (E, F) in nestling Willow (A, C, E) and Great Tits (B, D, F) in different caterpillar food availabilities.
**Figure S5.** Mean scaled environmental variances (CV_E_) of wing length (A, B), tail length (C, D), tarsus length (E, F) and body mass (G, H) in nestling Willow (A, C, E, G) and Great Tits (B, D, F, H) in different caterpillar food availabilities.

## Data Availability

Data available from the Dryad Digital Repository: https://doi.org/10.5061/dryad.xpnvx0kvh (Vatka et al., 2025).
