# Supplementary material for: Changing food availability and its effect on the heritability of offspring size in woodland passerine birds
Source: J Anim Ecol. 2025 Dec 4;95(3):497–508. doi: 10.1111/1365-2656.70204 (PMC12957738; doi:10.1111/1365-2656.70204)
Supplement: Supplementary file 1 — Table S1. Sample sizes for the Willow Tit, and numbers of individuals in pruned pedigrees. Table S2. Sample sizes for the Great Tit, and numbers of individuals in pruned pedigrees. Table S3. Model estimates from the random regression animal model for the tail length of the Willow Tit. Table S4. Model estimates from the random regression animal model for the tail length of the Great Tit. Figure S1. Additive genetic variance (A, B), error variance (C, D) and heritability estimates (E, F) of tail length in nestling Willow (A, C, E) and Great Tits (B, D, F) in different caterpillar food availabilities. Table S5. Model estimates from the random regression animal model for the tarsus length of the Willow Tit. Table S6. Model estimates from the random regression animal model for the tarsus length of the Great Tit. Figure S2. Additive genetic variance (A, B), error variance (C, D) and heritability estimates (E, F) of tarsus length in nestling Willow (A, C, E) and Great Tits (B, D, F) in different caterpillar food availabilities. Table S7. Model estimates from the random regression animal model for the body mass of the Willow Tit. Table S8. Model estimates from the random regression animal model for the body mass of the Great Tit. Figure S3. Additive genetic variance (A, B), error variance (C, D) and heritability estimates (E, F) of body mass in nestling Willow (A, C, E) and Great Tits (B, D, F) in different caterpillar food availabilities. Figure S4. Evolvabilities of tail length (A, B), tarsus length (C, D) and body mass (E, F) in nestling Willow (A, C, E) and Great Tits (B, D, F) in different caterpillar food availabilities. Figure S5. Mean scaled environmental variances (CVE) of wing length (A, B), tail length (C, D), tarsus length (E, F) and body mass (G, H) in nestling Willow (A, C, E, G) and Great Tits (B, D, F, H) in different caterpillar food availabilities. [file JANE-95-497-s001.pdf]

Supplementary material for Vatka, E., Orell, M., Rytkönen, S., & Merilä, J. (2025) Changing food availability and its effect on the heritability of offspring size in woodland passerine birds. Journal of Animal Ecology

Table S1. Sample sizes for the Willow Tit, and numbers of individuals in pruned pedigrees.

Response variable	N of nestlings	N of broods	N of years	N of females	N of males	N of measurers	N of individuals in a pedigree
Wing length	11 568	1 730	25	928	944	17	13 012
Tail length	11 567	1 730	25	928	944	17	13 011
Tarsus length	11 562	1 729	25	928	944	17	13 006
Body mass	11 567	1 730	25	928	944	-	13 011

Table S2. Sample sizes for the Great Tit, and numbers of individuals in pruned pedigrees.

Response variable	N of nestlings	N of broods	N of years	N of females	N of males	N of measurers	N of individuals in a pedigree
Wing length	7 554	1 131	21	869	875	24	9 064
Tail length	7 378	1 107	21	854	854	24	8 855
Tarsus length	7 521	1 129	21	869	874	24	9 030
Body mass	7 618	1 140	21	877	883	-	9 140

Table S3. Model estimates from the random regression animal model for the tail length of the Willow Tit. Food availability has been standardized by subtracting the mean and dividing by standard deviation.

Parameter	Posterior mode	95% HPD Interval		Effective sample size
Fixed effects				
Intercept	17.877	17.369	18.548	1000.0
Food availability	0.598	0.407	0.875	1000.0
Random effects				
V_Y σ^2_{year}	1.492	0.877	2.977	1000.0
V_{PE} σ^2_{brood}	5.906	5.218	6.432	1000.0
V_M $\sigma^2_{measurer}$	0.001	0.000	0.341	1000.0
V_A σ^2_A	1.543	0.677	2.659	897.3
$\sigma_{A,B}$	-0.083	-0.590	0.228	884.1
σ^2_B	0.429	0.000	0.767	1000.0
V_R $\sigma^2_{e,(-1.090\,-\,-0.621)}$	2.363	1.624	2.965	1000.0
$\sigma^2_{e,(-0.621\,-\,-0.157)}$	2.247	1.776	2.841	1000.0
$\sigma^2_{e,(-0.157\,-\,0.308)}$	1.934	1.383	2.457	941.7
$\sigma^2_{e,(0.308\,-\,0.772)}$	2.691	1.787	3.063	858.8
$\sigma^2_{e,(0.772\,-\,1.240)}$	1.974	1.120	2.540	870.5
$\sigma^2_{e,(1.240\,-\,1.700)}$	1.750	0.869	2.664	844.0
$\sigma^2_{e,(1.700\,-\,2.170)}$	1.024	0.192	2.273	863.8
$\sigma^2_{e,(2.170\,-\,2.630)}$	1.581	0.001	3.525	1000.0
$\sigma^2_{e,(2.630\,-\,3.090)}$	0.020	0.000	3.570	870.2
$\sigma^2_{e,(3.090\,-\,3.560)}$	0.012	0.000	2.980	889.7

σ^2_A= variance of random intercepts; σ^2_B= variance of random slopes; $\sigma_{A,B}$= covariance of random intercepts and slopes; σ^2_e= error variances in different food availability blocks

Table S4. Model estimates from the random regression animal model for the tail length of the Great Tit. Food availability has been standardized by subtracting the mean and dividing by standard deviation.

Parameter	Posterior mode	95% HPD Interval		Effective sample size
Fixed effects				
Intercept	22.211	21.081	23.316	1000.0
Food availability	1.317	0.989	1.810	1000.0
Random effects				
V_Y σ^2_{year}	3.856	1.805	8.582	1000.0
V_{PE} σ^2_{brood}	10.256	8.813	12.477	1000.0
V_M $\sigma^2_{measurer}$	0.708	0.140	3.634	1147.0
V_A σ^2_A	4.207	0.913	7.955	1113.0
$\sigma_{A,B}$	-1.589	-2.418	0.097	1147.0
σ^2_B	1.359	0.000	2.915	1000.0
V_R $\sigma^2_{e,(-1.760--1.310)}$	0.026	0.000	7.109	1143.0
$\sigma^2_{e,(-1.310--0.857)}$	3.102	1.458	7.799	1137.5
$\sigma^2_{e,(-0.857--0.406)}$	5.170	2.143	7.340	1191.8
$\sigma^2_{e,(-0.406-0.0449)}$	6.882	4.822	8.623	1149.4
$\sigma^2_{e,(0.0449-0.496)}$	7.058	5.218	8.907	1106.8
$\sigma^2_{e,(0.496-0.947)}$	6.427	4.763	8.216	1000.0
$\sigma^2_{e,(0.947-1.400)}$	7.035	4.813	8.390	1000.0
$\sigma^2_{e,(1.400-1.850)}$	3.202	2.239	5.909	863.6
$\sigma^2_{e,(1.850-2.300)}$	0.018	0.000	3.672	805.9
$\sigma^2_{e,(2.300-2.750)}$	0.018	0.000	7.630	832.5

σ^2_A= variance of random intercepts; σ^2_B= variance of random slopes; $\sigma_{A,B}$= covariance of random intercepts and slopes; σ^2_e= error variances in different food availability blocks

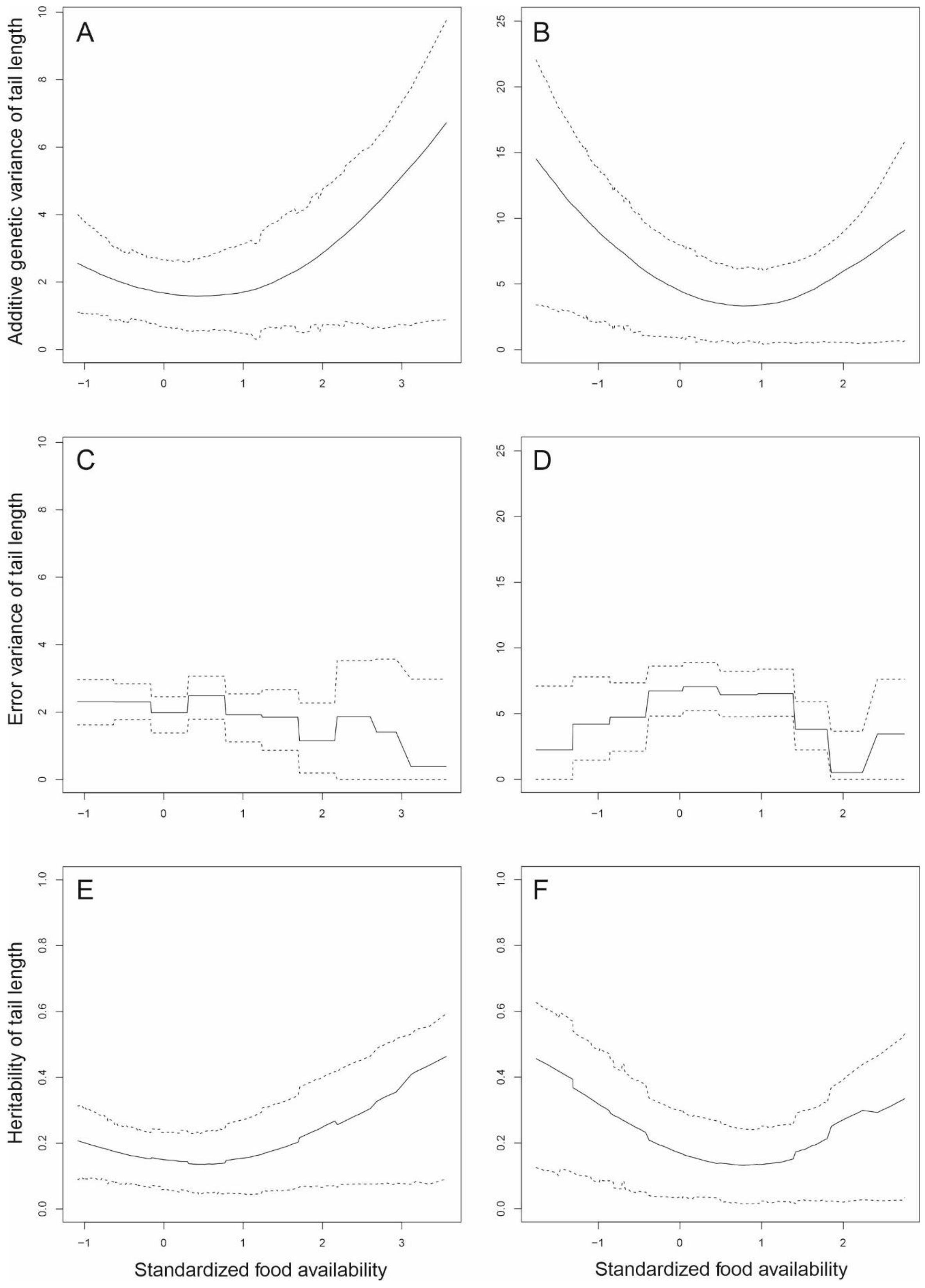

Figure S1. Additive genetic variance (A, B), error variance (C, D) and heritability estimates (E, F) of tail length in nestling Willow (A, C, E) and Great Tits (B, D, F) in different caterpillar food availabilities. Food availability has been standardized by subtracting the mean and dividing by standard deviation. Solid lines are median values of estimates for 1000 iterations, and dotted lines present HPD Intervals for the distributions of estimated values (please see Methods for details).

Table S5. Model estimates from the random regression animal model for the tarsus length of the Willow Tit. Food availability has been standardized by subtracting the mean and dividing by standard deviation.

Parameter		Posterior mode	95% HPD Interval		Effective sample size
Fixed effects					
Intercept		16.213	16.030	16.347	1118.0
Food availability		0.030	-0.015	0.060	1030.0
Random effects					
V_Y	σ^2_{year}	0.037	0.021	0.072	1000.0
V_{PE}	σ^2_{brood}	0.098	0.088	0.114	1000.0
V_M	$\sigma^2_{measurer}$	0.039	0.014	0.145	1000.0
V_A	σ^2_A	0.112	0.086	0.146	723.8
	$\sigma_{A,B}$	-0.001	-0.022	0.004	1000.0
	σ^2_B	0.000	0.000	0.020	1000.0
V_R	$\sigma^2_{e,(-1.090 - -0.622)}$	0.132	0.105	0.153	861.8
	$\sigma^2_{e,(-0.622 - -0.157)}$	0.169	0.143	0.181	964.7
	$\sigma^2_{e,(-0.157 - 0.307)}$	0.123	0.101	0.142	761.5
	$\sigma^2_{e,(0.307 - 0.772)}$	0.138	0.116	0.161	1000.0
	$\sigma^2_{e,(0.772 - 1.240)}$	0.132	0.105	0.160	1000.0
	$\sigma^2_{e,(1.240 - 1.700)}$	0.125	0.091	0.155	1000.0
	$\sigma^2_{e,(1.700 - 2.160)}$	0.123	0.091	0.162	1000.0
	$\sigma^2_{e,(2.160 - 2.630)}$	0.114	0.071	0.194	1212.8
	$\sigma^2_{e,(2.630 - 3.090)}$	0.114	0.032	0.165	1000.0
	$\sigma^2_{e,(3.090 - 3.560)}$	0.088	0.001	0.134	1000.0

σ^2_A= variance of random intercepts; σ^2_B= variance of random slopes; $\sigma_{A,B}$= covariance of random intercepts and slopes; σ^2_e= error variances in different food availability blocks

Table S6. Model estimates from the random regression animal model for the tarsus length of the Great Tit. Food availability has been standardized by subtracting the mean and dividing by standard deviation.

Parameter		Posterior mode	95% HPD Interval		Effective sample size
Fixed effects					
Intercept		19.222	19.026	19.396	1000.0
Food availability		0.106	0.041	0.180	1000.0
Random effects					
V_Y	σ^2_{year}	0.041	0.020	0.100	1000.0
V_{PE}	σ^2_{brood}	0.279	0.240	0.354	1000.0
V_M	$\sigma^2_{measurer}$	0.094	0.028	0.225	1000.0
V_A	σ^2_A	0.135	0.024	0.251	1000.0
	$\sigma_{A,B}$	0.000	-0.022	0.027	1000.0
	σ^2_B	0.000	0.000	0.022	1000.0
V_R	$\sigma^2_{e,(-1.720 - -1.270)}$	0.306	0.223	0.402	941.6
	$\sigma^2_{e,(-1.270 - -0.825)}$	0.329	0.253	0.383	1000.0
	$\sigma^2_{e,(-0.825 - -0.378)}$	0.328	0.243	0.384	1000.0
	$\sigma^2_{e,(-0.378 - 0.0701)}$	0.336	0.260	0.390	1000.0
	$\sigma^2_{e,(0.0701 - 0.518)}$	0.296	0.227	0.361	1099.2
	$\sigma^2_{e,(0.518 - 0.966)}$	0.235	0.155	0.293	1000.0
	$\sigma^2_{e,(0.966 - 1.410)}$	0.260	0.174	0.336	874.2
	$\sigma^2_{e,(1.410 - 1.860)}$	0.199	0.116	0.280	1115.3
	$\sigma^2_{e,(1.860 - 2.310)}$	0.133	0.000	0.198	1000.0
	$\sigma^2_{e,(2.310 - 2.760)}$	0.288	0.104	0.435	1000.0

σ^2_A= variance of random intercepts; σ^2_B= variance of random slopes; $\sigma_{A,B}$= covariance of random intercepts and slopes; σ^2_e= error variances in different food availability blocks

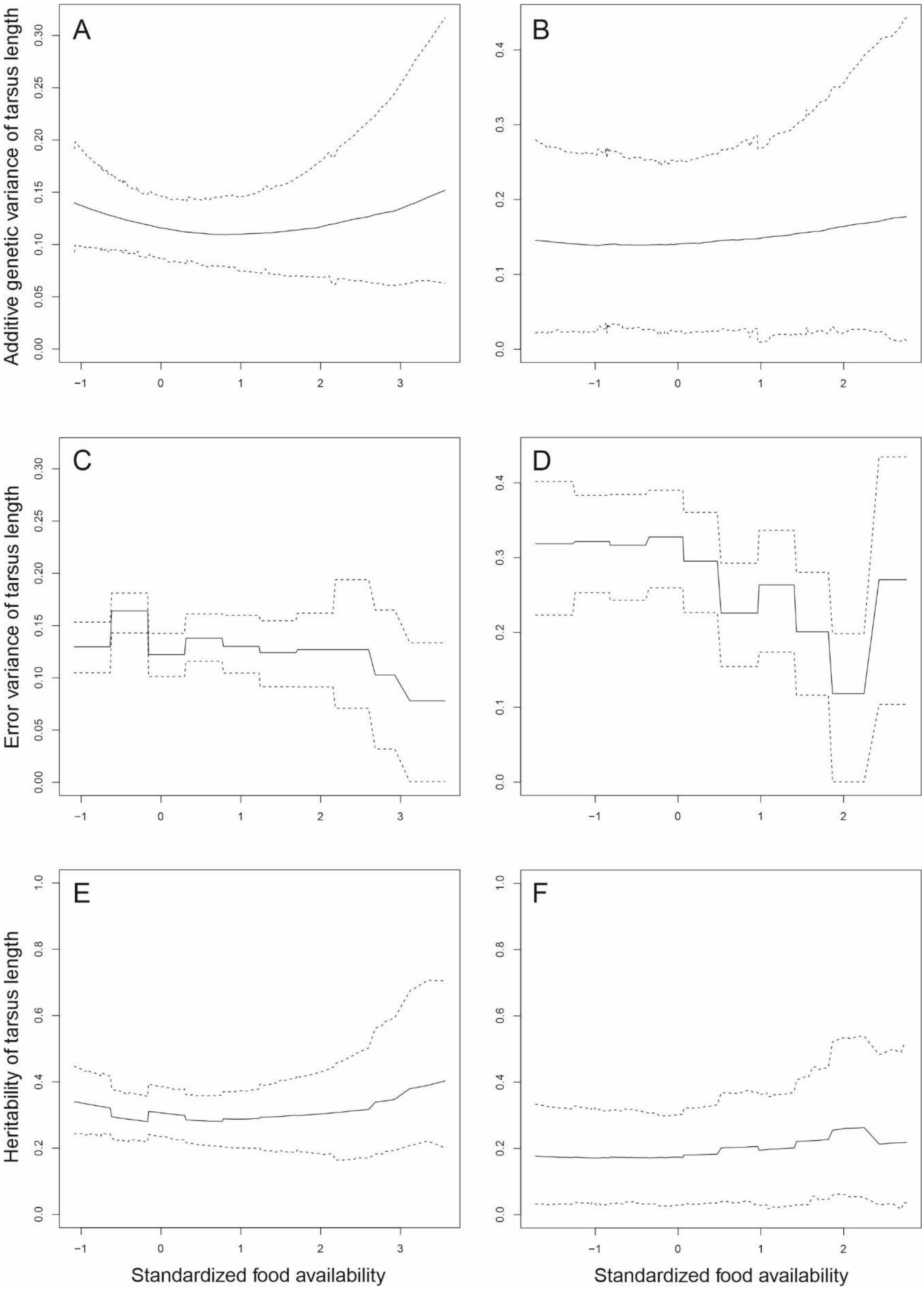

Figure S2. Additive genetic variance (A, B), error variance (C, D) and heritability estimates (E, F) of tarsus length in nestling Willow (A, C, E) and Great Tits (B, D, F) in different caterpillar food availabilities. Food availability has been standardized by subtracting the mean and dividing by standard deviation. Solid lines are median values of estimates for 1000 iterations, and dotted lines present HPD Intervals for the distributions of estimated values (please see Methods for details).

Table S7. Model estimates from the random regression animal model for the body mass of the Willow Tit. Food availability has been standardized by subtracting the mean and dividing by standard deviation.

Parameter		Posterior mode	95% HPD Interval		Effective sample size
Fixed effects					
Intercept		10.970	10.823	11.098	1000.0
Food availability		0.053	-0.005	0.113	1000.0
Random effects					
V_Y	σ^2_{year}	0.114	0.057	0.200	1000.0
V_{PE}	σ^2_{brood}	0.280	0.251	0.322	1000.0
V_A	σ^2_A	0.269	0.194	0.354	1000.0
	$\sigma_{A,B}$	-0.053	-0.078	-0.000	1000.0
	σ^2_B	0.040	0.000	0.065	1000.0
V_R	$\sigma^2_{e,(-1.090 - -0.621)}$	0.283	0.215	0.339	1000.0
	$\sigma^2_{e,(-0.621 - -0.157)}$	0.304	0.250	0.346	1000.0
	$\sigma^2_{e,(-0.157 - 0.308)}$	0.275	0.228	0.333	1000.0
	$\sigma^2_{e,(0.308 - 0.772)}$	0.232	0.182	0.286	1096.0
	$\sigma^2_{e,(0.772 - 1.240)}$	0.244	0.189	0.307	1000.0
	$\sigma^2_{e,(1.240 - 1.700)}$	0.305	0.240	0.383	1000.0
	$\sigma^2_{e,(1.700 - 2.170)}$	0.181	0.118	0.248	1000.0
	$\sigma^2_{e,(2.170 - 2.630)}$	0.175	0.082	0.290	1000.0
	$\sigma^2_{e,(2.630 - 3.090)}$	0.002	0.000	0.140	1000.0
	$\sigma^2_{e,(3.090 - 3.560)}$	0.307	0.155	0.502	1220.0

σ^2_A= variance of random intercepts; σ^2_B= variance of random slopes; $\sigma_{A,B}$= covariance of random intercepts and slopes; σ^2_e= error variances in different food availability blocks

Table S8. Model estimates from the random regression animal model for the body mass of the Great Tit. Food availability has been standardized by subtracting the mean and dividing by standard deviation.

Parameter		Posterior mode	95% HPD Interval		Effective sample size
Fixed effects					
Intercept		15.758	15.559	16.030	1000.0
Food availability		0.345	0.145	0.484	1000.0
Random effects					
V_Y	σ^2_{year}	0.150	0.052	0.451	1000.0
V_{PE}	σ^2_{brood}	2.587	2.268	3.099	1000.0
V_A	σ^2_A	1.277	0.530	2.219	874.4
	$\sigma_{A,B}$	-0.001	-0.295	0.180	1117.1
	σ^2_B	0.002	0.000	0.365	1000.0
V_R	$\sigma^2_{e,(-1.730 - -1.280)}$	0.006	0.000	1.378	1000.0
	$\sigma^2_{e,(-1.280 - -0.827)}$	1.318	0.634	1.736	1000.0
	$\sigma^2_{e,(-0.827 - -0.378)}$	1.567	1.014	2.085	1000.0
	$\sigma^2_{e,(-0.378 - 0.072)}$	1.417	0.908	1.856	905.5
	$\sigma^2_{e,(0.072 - 0.522)}$	1.171	0.677	1.562	830.8
	$\sigma^2_{e,(0.522 - 0.971)}$	1.105	0.650	1.567	1000.0
	$\sigma^2_{e,(0.971 - 1.420)}$	1.070	0.600	1.520	1000.0
	$\sigma^2_{e,(1.420 - 1.870)}$	0.567	0.261	1.168	887.4
	$\sigma^2_{e,(1.870 - 2.320)}$	0.005	0.000	0.724	1000.0
	$\sigma^2_{e,(2.320 - 2.770)}$	0.006	0.000	0.945	1000.0

σ^2_A= variance of random intercepts; σ^2_B= variance of random slopes; $\sigma_{A,B}$= covariance of random intercepts and slopes; σ^2_e= error variances in different food availability blocks

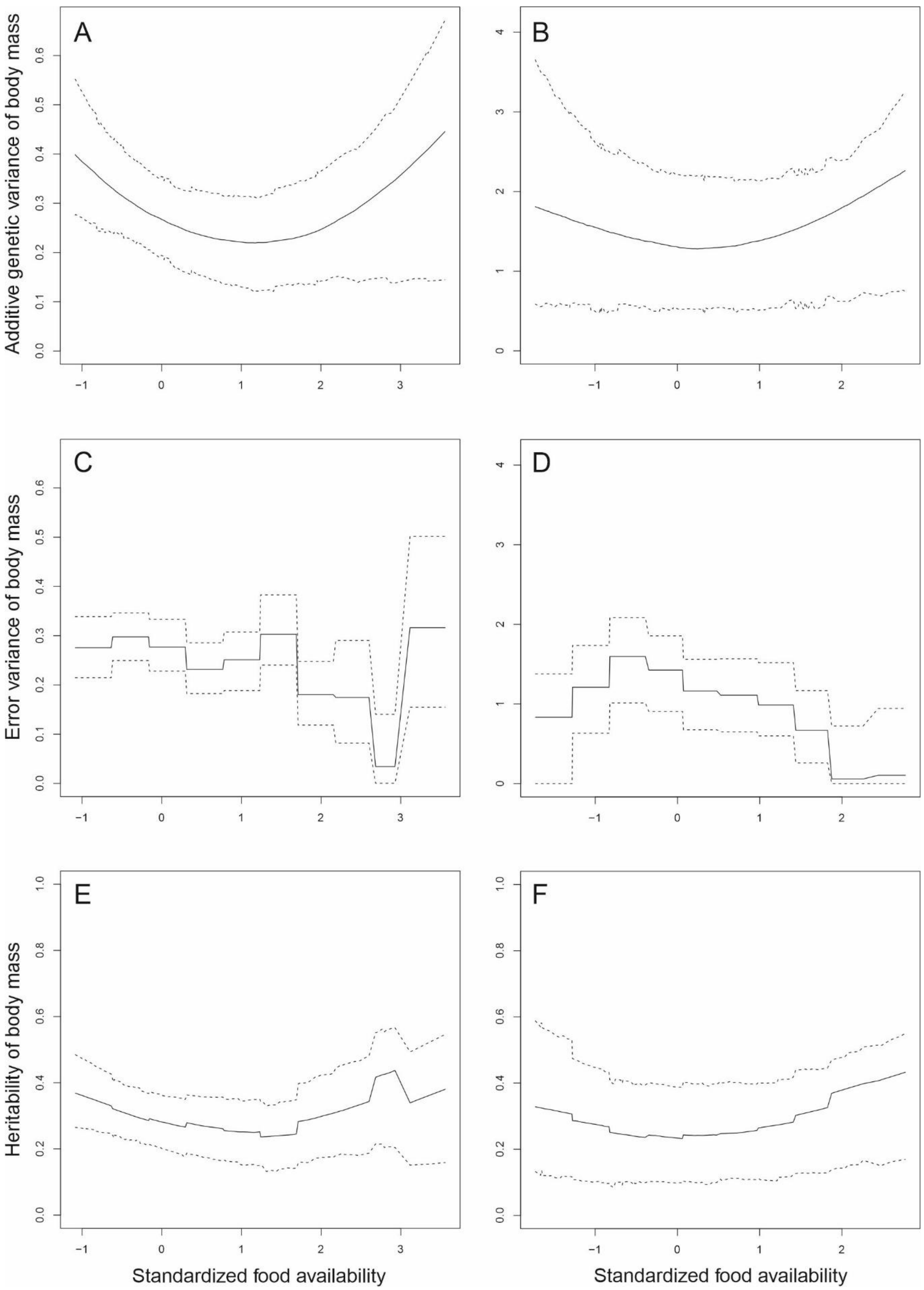

Figure S3. Additive genetic variance (A, B), error variance (C, D) and heritability estimates (E, F) of body mass in nestling Willow (A, C, E) and Great Tits (B, D, F) in different caterpillar food availabilities. Food availability has been standardized by subtracting the mean and dividing by standard deviation. Solid lines are median values of estimates for 1000 iterations, and dotted lines present HPD Intervals for the distributions of estimated values (please see Methods for details).

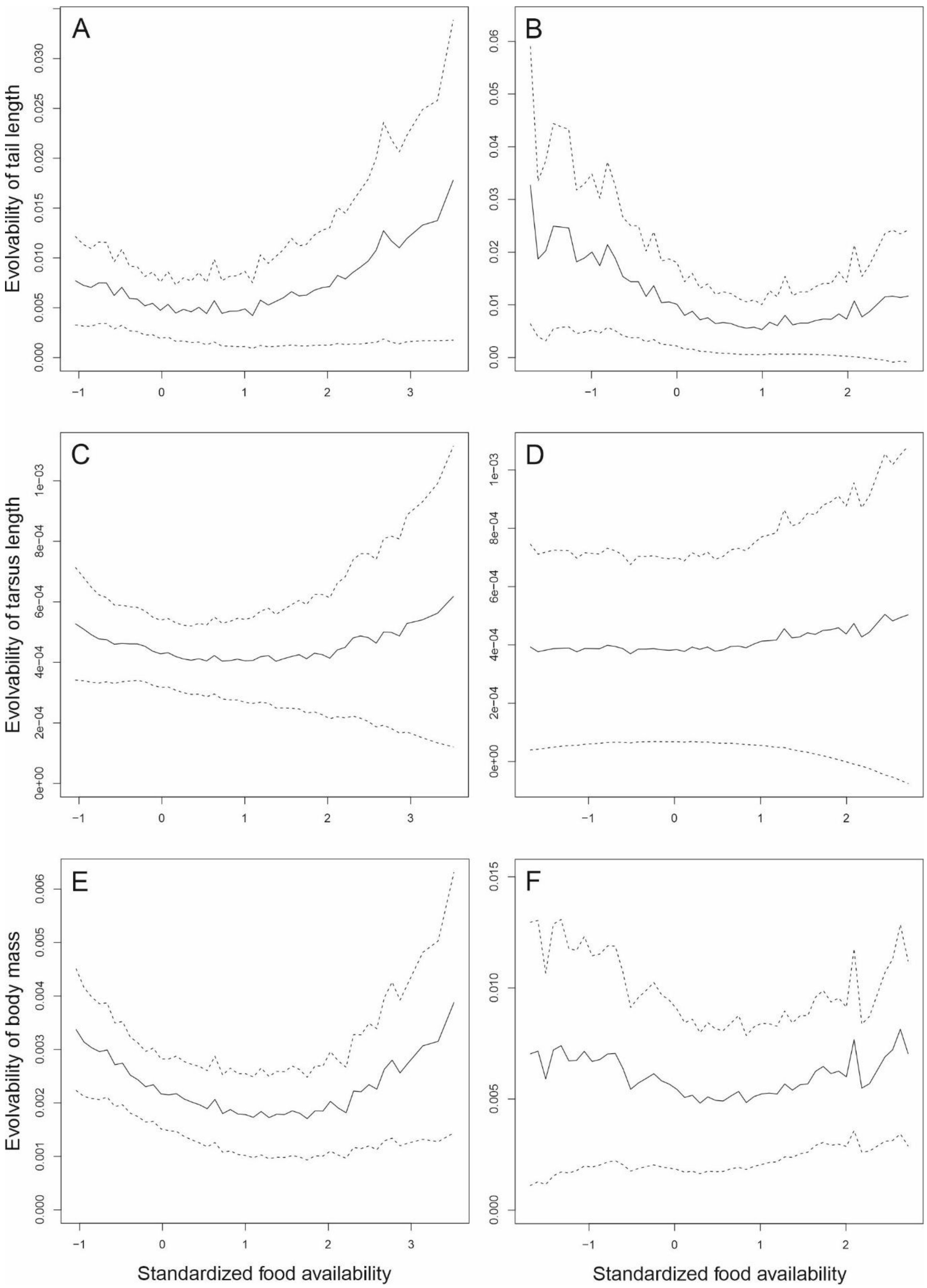

Figure S4. Evolvabilities of tail length (A, B), tarsus length (C, D) and body mass (E, F) in nestling Willow (A, C, E) and Great Tits (B, D, F) in different caterpillar food availabilities. Food availability has been standardized by subtracting the mean and dividing by standard deviation. Solid lines are mean values of estimates, and dotted lines present 95% confidence intervals (please see Methods for details).

Calculations of mean scaled environmental variances (CV_E)

Similarly to estimating evolvabilities, we also estimated mean scaled environmental variances (CV_E) for varying food availability values using the formula

$$CV_{E_k} = \frac{V_{E_k}}{\bar{x}_k^2} = \frac{V_Y + V_{PE} + V_{R_k}}{\bar{x}_k^2} \qquad \text{(S1)}$$

where $\bar{x}_k$ is the trait mean in a food availability *k*. To calculate these mean trait values, we divided food availabilities into 50 equal-interval groups and calculated mean values (and standard errors) of trait values for each food availability group. The uncertainty of the CV_E estimates were calculated as 95% confidence intervals, considering uncertainties of both V_E and $\bar{x}$:

$$95\%\ CI_k = 1.96 * \sqrt{\frac{SE(V_{E_k})^2}{V_{E_k}} + (2\frac{SE(\bar{x}_k)}{\bar{x}_k})^2} * CV_{E_k} \qquad \text{(S2)}$$

CV_E values for nestlings' body size traits with varying food availabilities are presented in Fig. S5. Their variation mostly reflects the estimated variation in residual variance.

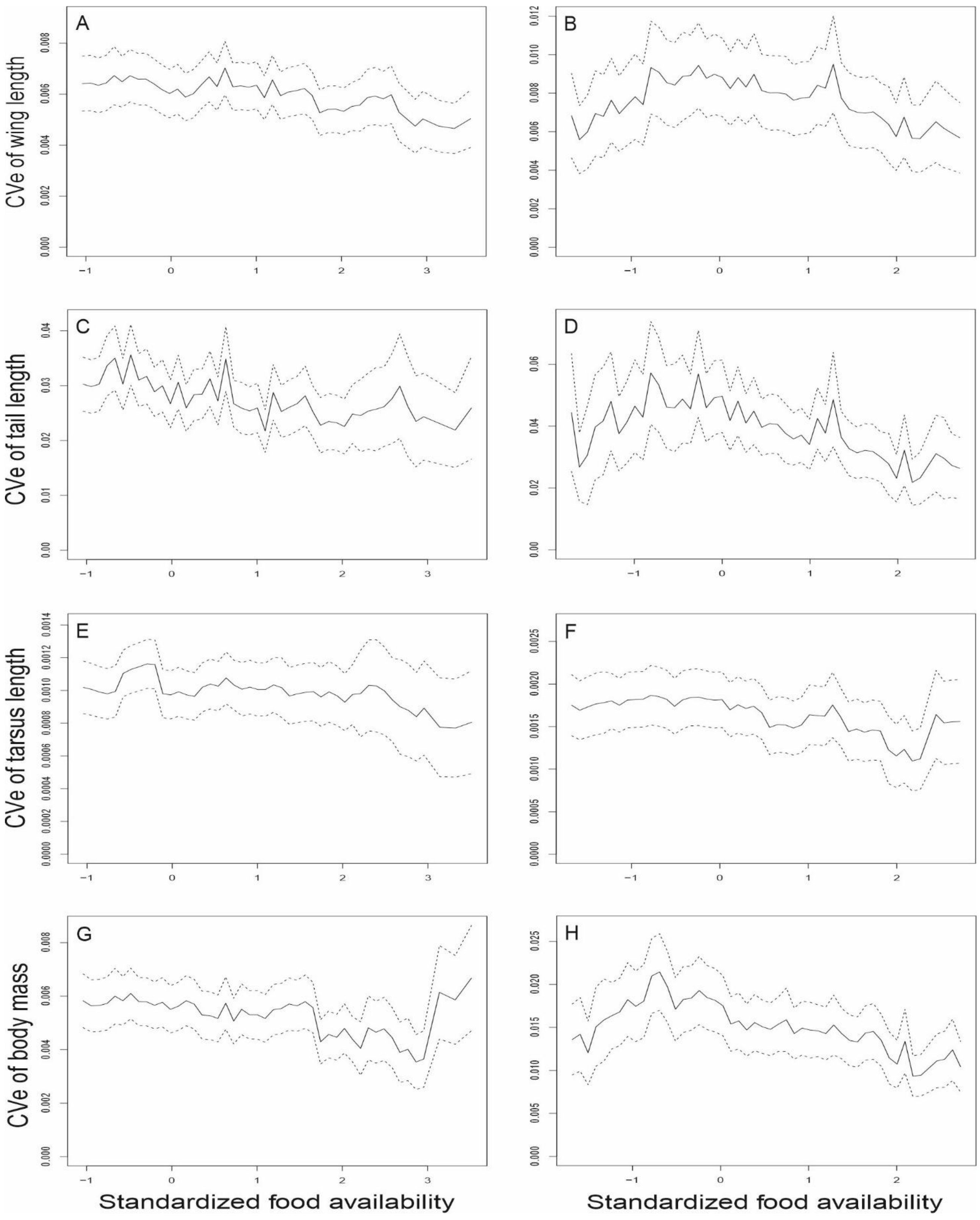

Figure S5. Mean scaled environmental variances (CV_E) of wing length (A, B), tail length (C, D), tarsus length (E, F) and body mass (G, H) in nestling Willow (A, C, E, G) and Great Tits (B, D, F, H) in different caterpillar food availabilities. Food availability has been standardized by subtracting the mean and dividing by standard deviation. Solid lines are mean values of estimates, and dotted lines present 95% confidence intervals.
